# Chronic Joint Pain in a Young Adult With Cystic Fibrosis

**DOI:** 10.7759/cureus.17229

**Published:** 2021-08-16

**Authors:** Bhavini B Prajapati, Alexandra Filippi, Edmund H Sears

**Affiliations:** 1 Internal Medicine/Pediatrics, Maine Medical Center, Portland, USA; 2 Pulmonary and Critical Care Medicine, Maine Medical Center, Portland, USA; 3 Pulmonary Medicine, Maine Medical Center, Portland, USA

**Keywords:** hypertrophic pulmonary osteoarthropathy, cystic fibrosis, cf associated joint pain, trikafta, homozygous f508 del mutation

## Abstract

A 25-year-old male with end-stage cystic fibrosis (CF) with genotype F508del/F508del presented to the clinic complaining of bilateral knee and ankle pain. He had severe lung disease (forced expiratory volume 1 {FEV1} 19% of predicted), chronic colonization with achromobacter, malnutrition, and CF-related diabetes. On physical examination, he was found to have bilateral knee swelling as well as pain on flexion and extension of the wrists and ankles without erythema or warmth. He was empirically started on prednisone and tramadol; however, at a three-month follow-up visit, he remained symptomatic. He was sent for a whole-body bone scan, which was consistent with hypertrophic pulmonary osteoarthropathy (HPOA). He was started on highly effective modulator therapy with elexacaftor/tezacaftor/ivacaftor and symptoms spontaneously resolved without further intervention.

## Introduction

Hypertrophic pulmonary osteoarthropathy (HPOA) is a syndrome characterized by digital clubbing, periostosis, and joint swelling. Chronic lung disease, such as that seen in patients with cystic fibrosis (CF), is a frequent association and implicated in the pathophysiology [1,2]. This condition is increasingly being recognized as a cause of chronic joint pain in patients with late-stage CF. Nuclear medicine bone scan is a sensitive diagnostic test that can identify HPOA. Treatment of the underlying condition usually results in the improvement of joint symptoms.

This article was previously published as a meeting abstract at the Northern New England Cystic Fibrosis Consortium on August 27, 2020. 

## Case presentation

A 25-year-old male with end-stage CF with genotype F508del/F508del presented to the clinic complaining of chronic bilateral knee and ankle pain. He had severe lung disease with forced expiratory volume 1 (FEV1) 19% predicted. He had previously undergone a childhood liver transplant for CF liver disease and remained on tacrolimus. He also had CF-related diabetes, pancreatic insufficiency, and malnutrition. His current medications included dornase alfa, albuterol MDI prn, and pancreatic enzymes, however, he was not on a cystic fibrosis transmembrane conductance regulator (CFTR) modulator at the time. The patient resided in rural Maine although he denied any recent insect bites. He was not sexually active at the time of his initial presentation. 

**Figure 1:**
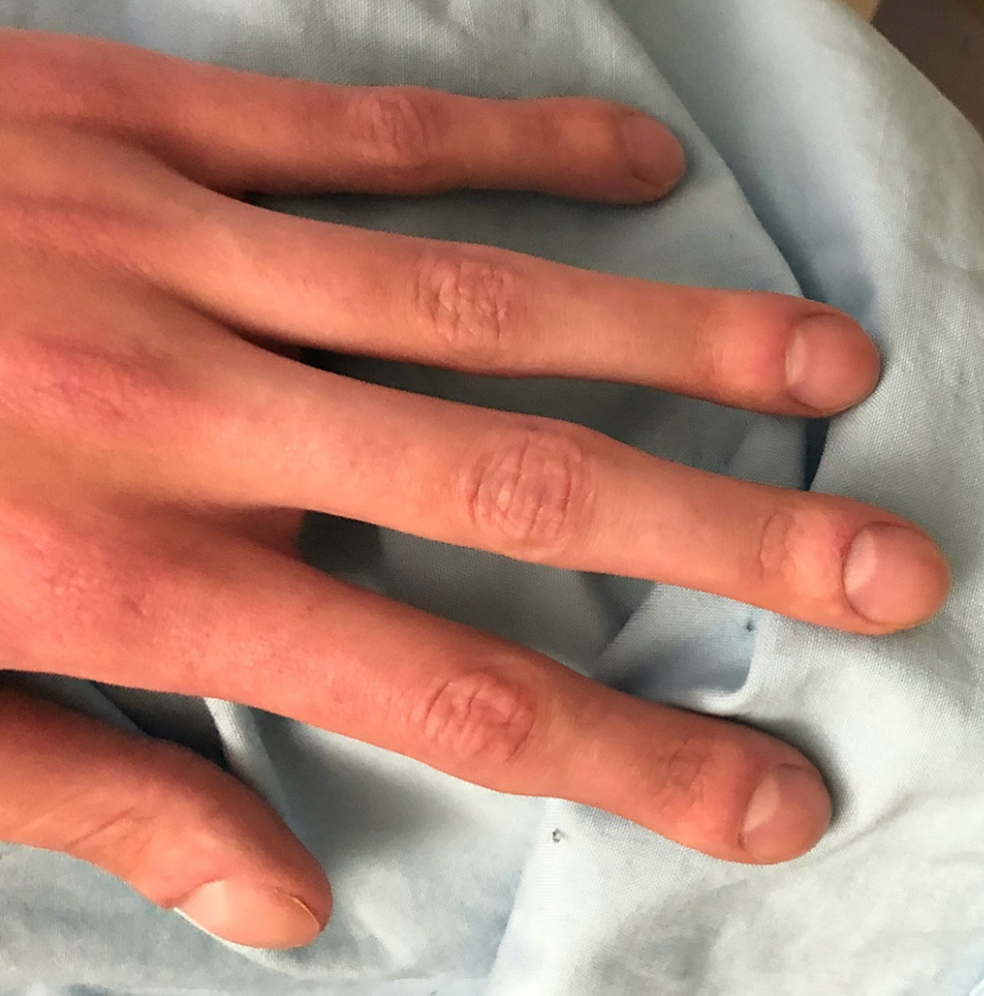
Swollen DIP and PIP joints from above (stage 3 clubbing) DIP: distal interphalangeal; PIP: proximal interphalangeal

On physical examination at his initial presentation, his vitals were within normal limits (temp 36.6°C, HR 105, BP 118/74, SpO_2_ 91%). He was found to have swelling of bilateral knees as well as pain on flexion and extension of the wrists and ankles. None of his affected joints were erythematous or warm. He had no joint deformities and no frozen joints. He had grade 3 clubbing in his hands bilaterally (Figure 1).

**Figure 2:**
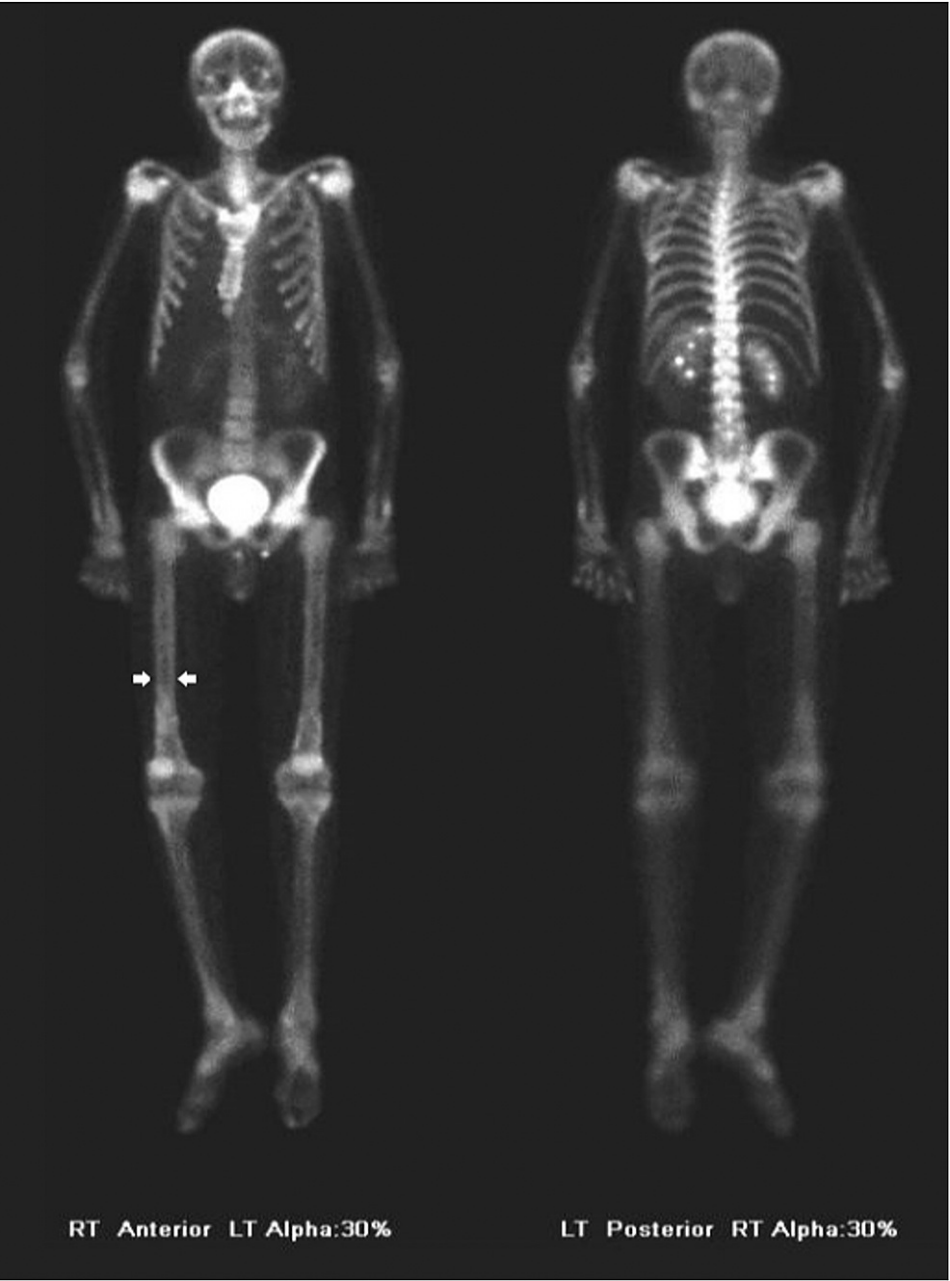
Nuclear bone scan (arrows indicating tram-tracking sign)

He was started empirically on 10 mg prednisone for a suspected diagnosis of CF arthropathy and was given ibuprofen and tramadol for pain relief. He had improvement in his joint swelling but continued to have frequent joint pain. At a follow-up visit, prednisone was decreased to 5 mg due to hyperglycemia and he was started on highly effective modulator therapy with elexacaftor/tezacaftor/ivacaftor (Trikafta®). A whole-body bone scan was obtained (Figure 2). The bone scan showed evidence of periostosis and thus the diagnosis of HPOA was made.

## Discussion

Cystic fibrosis is a multisystem disease caused by impaired chloride ion channel function that can result in chronic hypoxia. The musculoskeletal system is one of the many end-organs affected by this disease, and joint pain is not an uncommon complaint in patients with CF. The differential for young adults with joint pain should include septic arthritis including those from sexually transmitted diseases, crystalline arthropathy, Lyme arthritis, inflammatory arthritis, rheumatologic disease, and post-traumatic osteoarthritis. In a patient with CF, CF arthropathy, fluoroquinolone tendonitis, and HPOA are reasonable differential diagnoses as well. 

Clinical suspicion for specific disease processes can guide the initial management and diagnostic workup. Arthrocentesis is helpful in evaluating for infectious, crystalline, and inflammatory causes of joint effusion, which is usually sterile in patients with HPOA. The pattern of radiologic abnormalities can lend support to an underlying mechanism of joint injury and further assist in ascertaining the causative etiology. Autoimmune serology in CF patients is often difficult to interpret as there is limited data on the clinical significance of the tests. Several studies have demonstrated low titer elevations of anti-cyclic citrullinated peptide antibodies, rheumatoid factor, anti-nuclear antibody test (ANA), and positive anti-neutrophil cytoplasmic antibodies pattern that do not have known disease associations to date [2-4]. 

CF arthropathy (CFA) is an oligoarthritis that often has a relapsing and remitting course and is felt to be due to chronic inflammation either via the formation of immune complexes or from molecular mimicry to antigens produced by chronic infection, particularly *Pseudomonas aeruginosa* [5]. It is often treated with low-dose prednisone or other immunosuppressants such as hydroxychloroquine. In contrast, HPOA shares some of these clinical features but is differentiated by the presence of bony pain and periostosis of tubular bones radiographically [6].

Previously, HPOA was thought to be rare in patients with cystic fibrosis. The average age of diagnosis of HPOA is generally later than CFA and has been associated with lower lung function [1]. As the life expectancy of patients with CF has increased, there has also been growing recognition of this condition. 

HPOA is also associated with a number of different diseases ranging from cyanotic heart and chronic pulmonary disorders to various hematologic, hepatic, and gastrointestinal conditions [7-9]. HPOA has been closely tied to poor respiratory status in chronic lung diseases including CF [2]. Central to the proposed pathophysiology of HPOA in chronic lung diseases is intra-or-extrapulmonary shunting of blood bypassing the lungs [10]. While the exact mechanism has not been well delineated several hypotheses have been proposed. It is suspected that vascular endothelial growth factor (VEGF), a cytokine that is known to play a role in vascular hyperplasia, edema formation, and new bone formation, may also play a role in HPOA [10,11]. There is also a rare primary form of HPOA, which is known as pachydermoperiostosis. In this case, a role has been described for circulating levels of prostaglandin E2 [12]. 

HPOA has a variable clinical presentation. Some patients may describe no joint or bony pain and may have only severe digital clubbing, which is the most common manifestation on physical examination. In others, pain and swelling of one or more joints may be the predominant symptoms and warrant broader diagnostic considerations [13]. The distribution and acuity of joint involvement as well as other predisposing conditions or family history can narrow the differential diagnosis.

Periostitis is the radiographic hallmark of HPOA and manifests along the shafts of tubular bones. As the disease progresses the periostitis becomes more prominent and extends to involve the epiphyses [14]. X-rays of the long bone, therefore, show metaphyseal and diaphyseal smooth periosteal reaction. Typically, there is symmetrical involvement of the appendicular skeleton with a wide distribution [14]. In contrast to other disorders, joint involvement in HPOA is characterized by the presence of synovial effusion without evidence of joint space narrowing, erosions, or periarticular osteopenia [14]. MR imaging typically exhibits periosteal reaction low-to-intermediate signal intensity on T1-weighted images and low signal intensity on T2-weighted images. Its appearance on MR often correlates with the radiographic findings and can manifest as simple periosteal elevation or laminated periosteal reaction [14]. Radionuclide bone scanning is more sensitive for the detection of HPOA than radiography alone. On nuclear medicine (NM) bone scan, there is a classic “tram-track sign” that results from the symmetric linear increase in tracer accumulation along diaphyseal and metaphyseal surfaces of long bones, as was seen in this case [14,15]. 

Symptoms often remit with the treatment of the underlying disease. For symptomatic patients, non-steroidal anti-inflammatory drugs (NSAIDs) are the treatment of choice with ibuprofen and indomethacin being the favored agents [16]. Steroids have also been shown to be beneficial, likely due to their anti-inflammatory effects. Opioids may occasionally be used for refractory symptoms [17]. Bisphosphonates or unilateral vagotomy may be rare alternative options as well. Vagotomy was one of the earliest proposed therapies, however, it is now rarely used due to its varying degrees of success and invasive nature [18]. Another promising treatment that has been investigated is octreotide, which is a somatostatin analog and controls the growth and secretions of pituitary adenomas in acromegaly [18]. Bisphosphonates inhibit osteoclastic bone resorption and have been demonstrated to reduce pain in patients with advanced malignancy [17]. Intravenous pamidronate has been described as an effective treatment for pain associated with HPOA [19]. The mechanism behind the action of bisphosphonates remains somewhat unclear. 

In our case, after starting elexacaftor/tezacaftor/ivacaftor (Trikafta®), a highly effective CFTR modulator, the patient has a significant improvement in his overall lung function and CF symptoms. His joint complaints dramatically lessened over the next few months as well. We suspect direct treatment of his underlying CF played the largest role in the resolution of his skeletal pain due to HPOA.

## Conclusions

Joint pain is a common complaint in patients with cystic fibrosis. The differential diagnosis is broad and includes a variety of infectious and inflammatory conditions prevalent in young adults. CFA and HPOA should be considered in patients with longstanding CF; CFA tends to present earlier in the disease course and does not correlate as strongly with the severity of lung disease. Physical examination findings of clubbing and joint swelling along with imaging showing periostosis confirm the diagnosis of HPOA. The mainstay of management is the treatment of the underlying condition, which in this case was the addition of a highly effective CFTR modulator.
